# Wnt Signaling Enhances Neurogenesis and Improves Neurological Function after Focal Ischemic Injury

**DOI:** 10.1371/journal.pone.0040843

**Published:** 2012-07-17

**Authors:** Adi Shruster, Tali Ben-Zur, Eldad Melamed, Daniel Offen

**Affiliations:** Laboratory of Neuroscience, Felsenstein Medical Research Center, Sackler Faculty of Medicine, Tel Aviv University, Israel; Massachusetts General Hospital/Harvard Medical School, United States of America

## Abstract

Stroke potently stimulates cell proliferation in the subventricular zone of the lateral ventricles with subsequent neuroblast migration to the injured striatum and cortex. However, most of the cells do not survive and mature. Extracellular Wnt proteins promote adult neurogenesis in the neurogenic niches. The aim of the study was to examine the efficacy of Wnt signaling on neurogenesis and functional outcome after focal ischemic injury. Lentivirus expressing Wnt3a-HA (LV-Wnt3a-HA) or GFP (LV-GFP) was injected into the striatum or subventricular zone of mice. Five days later, focal ischemic injury was induced by injection of the vasoconstrictor endothelin-1 into the striatum of the same hemisphere. Treatment with LV-Wnt3a-HA into the striatum significantly enhanced functional recovery after ischemic injury and increased the number of BrdU-positive cells that differentiated into mature neurons in the ischemic striatum by day 28. Treatment with LV-Wnt3a-HA into the subventricular zone significantly enhanced functional recovery from the second day after injury and increased the number of immature neurons in the striatum and subventricular zone. This was accompanied by reduced dissemination of the neuronal injury. Our data indicate that Wnt signaling appears to contribute to functional recovery after ischemic injury by increasing neurogenesis or neuronal survival in the striatum.

## Introduction

Focal brain ischemia stimulates the proliferation of neuronal precursor cells in the subventricular zone (SVZ), followed by migration of neuroblasts into the ischemic regions [1]. However, although many neuroblasts reach the injured striatum, very few differentiate into mature neurons [2]. Given that increased neurogenesis around ischemic lesions improves clinical outcome [1], [3], these findings raise the possibility that enhancing neuronal differentiation and survival could serve as a therapeutic approach to stroke.

For an outside factor to support long-term neuronal regeneration, its continuous or repeated administration is necessary. The administration of any therapeutic protein is problematic because it almost never passes the blood-brain barrier and the half-life is relatively short. Gene therapy may be a good alternative, as a single injection is sufficient for local production of the relevant protein for a long period.

Wnt proteins are extracellular factors that play important roles in the developed and mature central nervous system. They regulate the proliferation of neural progenitor cells and their differentiation to neurons in the subventricular and subgranular zones [4]. Moreover, the Wnt signaling pathway is an obligate component of neural progenitor cell differentiation into neurons [5]. However, whether Wnt signaling can create the appropriate environment for neuronal differentiation and survival outside the classic neurogenic niche remains unclear, as does its potential contribution to clinical improvement after ischemic injury.

The aim of the present study was to investigate the effect of lentiviral-mediated Wnt3a (LV-Wnt3a-HA) gene transfer on neural progenitor cell proliferation and neurogenesis in the striatum after focal ischemic injury in a mouse model.

## Materials and Methods

### Cloning Lentiviral Vectors

The lentiviral vectors LV-Wnt3a-HA and LV-GFP were constructed using the ViraPower Promoterless Lentiviral Gateway® Kit (Invitrogen, San Diego, CA, USA) according to the manufacturer’s protocol. The cytomegalovirus (CMV) promoter from pIRES2/AcGFP1 cDNA (Clontech, Palo Alto, CA, USA) was cloned into pENTR™5′-TOPO® (Invitrogen). The Wnt3a-HA gene (bearing the HA tag) from the pBSWnt-3aHA cDNA (Addgene, Cambridge, MA, USA) and the AcGFP1 gene from the pIRES2/AcGFP1 cDNA (Clontech) served as the expression genes. The constructs were cloned into the pCR®8/GW/TOPO® (Invitrogen). The final expression constructs were obtained by recombination of the entry clone harboring the CMV promoter, the entry clone harboring the expression gene of interest, and pLenti6/R4R2/V5-DEST (Invitrogen).

### High-Titer Lentiviral Preparation

The expression constructs were co-transfected with DNA mixture containing transfer plasmid, packaging plasmid (encoding viral Gag and Pol proteins, pCMVΔR8.91), envelope plasmid (encoding the envelope of vesicular stomatitis virus, pCI), and LipofectAMINE 2000 (invitrogen). To obtain high-titer viral stocks, at 48 and 72 hours after transfection, the medium was collected, cleared by low-speed centrifugation, filtered through a 0.45-µm-pore-size filter, and ultracentrifuged at 25,000 rpm for 2 hours at 4°C (Beckman Coulter, Inc., Palo Alto, CA, USA). Viral titers were determined by transduction of HeLa cells with serial dilutions of the viral supernatant and colony counting after blasticidin selection (4 ng/mL, Invitrogen) using crystal violet staining (Sigma-Aldrich, St. Louis, MO, USA). LV stock titers were expressed as transducing units (TU) per milliliter and ranged in the order of 10^9^ TU ml^−1^. HeLa cells were commercially acquired from ATCC CCL-2 (Manassas, USA).

### In Vitro Validation of Lentiviral Vectors

The ability of the LV-Wnt3a-HA vectors to express Wnt3a was assessed by Western blot analysis and immunocytochemistry. HeLa cells were treated with LV-Wnt3a-HA or with non-related-gene-expressing viruses. For Western blot analysis, the cells were harvested 48 h post infection. Protein extraction and Western blotting were performed, as previously described [6]. The membrane was probed with mouse monoclonal anti-HA antibody clone 11 (1∶1000, Covance, Berkeley, CA, USA), mouse anti-active-β-catenin (1∶500; Millipore, Billerica, MA, USA) and rabbit anti-emerin (1∶5,000; Santa Cruz Santa Cruz, CA, USA). Secondary detection was carried out using two infrared fluorescent dye conjugated goat antibodies: IRDye® 800 CW and IRDye® 680 LT (LI-COR Biosiences, Lincoln, Nebraska, 1∶200). The membrane was imaged on an Odyssey infrared scanner with sensitivity of six in both the 700 and 800 nm wavelength channels. Data were acquired by using Odyssey software.

For fluorescent microscope visualization of Wnt3a-HA, Hela cells were grown on plates for 48 hours, fixed with 4% paraformaldehyde (PFA), and incubated with mouse monoclonal anti-HA antibody clone 11 (1∶1000, Covance, Berkeley, CA, USA) and goat anti-mouse Alexa 488 secondary antibody (1∶500, Molecular Probes, Invitrogen). Nuclear DNA was stained with DAPI (1∶200, Sigma-Aldrich).

### Ethics Statement

Animals were used in full compliance with the National Institutes of Health/Institutional Animal Care and Use Committee guidelines. All animal studies were approved by the Animal Care and Use Committee of Tel Aviv University under protocol # M-11-002.

### Animals

Adult male C57BL/6 mice (Harlan, Jerusalem, Israel), 8 weeks old, were used in this study. All mice were maintained at a mean room temperature of 23±2°C on a 12-hour/12-hour light/dark cycle. Food and water were provided *ad libitum*.

### Lentiviral Injection

**Figure 1:**
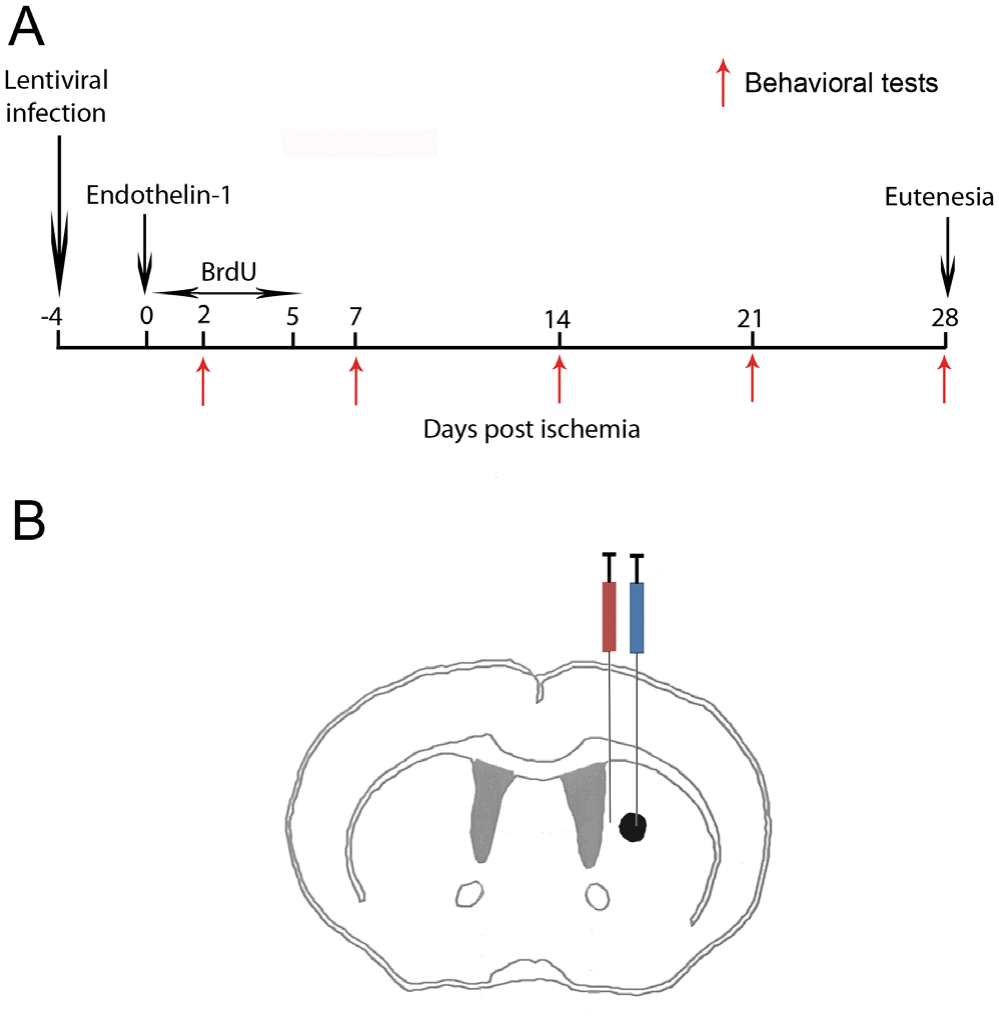
Schematic representation of the experimental design. A. Experimental protocol. B. Diagram of the brain section. Lentiviral vector was injected into either the striatum (blue syringe) or SVZ (red syringe). The ischemic area in the striatum is circled in black.

Mice were anesthetized with a mixture of ketamine-xylazine, and after proper sterilization and wellness procedures, were placed in a stereotaxic frame (Stoelting, Wood Dale, IL, USA). LV-Wnt3a-HA was injected into the right striatum (n = 12) or the right SVZ (n = 12) (1 µl, infusion rate of 0.3 µl/min) using the following coordinates (relative to bregma and according to the atlas of Paxinos and Watson [7]): striatum: +0.5 mm anteroposterior, +1.5 mm mediolateral, and −2.9 mm dorsoventral; SVZ: +0.5 mm anteroposterior, +1 mm mediolateral, −2.9 mm dorsoventral (Figure 1A–B). LV-GFP (n = 12) and phosphate buffered saline (PBS) (n = 8) were used as controls for each injection site. Additionally, two groups of mice were injected with LV-Wnt3a-HA (n = 5) or LV-GFP (n = 5) into the SVZ for short-term histochemical analysis (2 days after ischemia).

### Focal Ischemia Induction

Five days after LV injection (Figure 1A), 1 µl of the vasoconstrictor endothelin-1 (1 mg/ml dissolved in ddH_2_O; Calbiochem, La Jolla, CA, USA) was injected into the right striatum: +0.5 mm anterioposterior, +1.9 mm and +1.5 mm mediolateral, −2.9 mm dorsoventral (total volume 2 µl; infusion rate 0.125 µl/min) (Figure 1A,B) of all mice.

### Proliferation Labeling

To examine the fate of the constitutively proliferating cell population, mice were injected intraperitoneally with 100 mg/kg body weight of bromodeoxyuridine (BrdU; Sigma-Aldrich), 1 injection per day for 5 consecutive days after the focal ischemia induction injection (Figure 1A). The groups injected with LV into the SVZ for short-term histochemical analysis, were injected with 50 mg/kg 5-ethynyl-2′-deoxyuridine (EdU) (Invitrogen) for 2 consecutive days after focal ischemia induction.

### Behavioral Tests

Two behavioral tests were performed at 2, 7, 14, 21, and 28 days after ischemia induction (Figure 1A).

#### Cylinder test

The 10-minute cylinder test assesses the symmetry of forelimb use and was performed as previously described [8]. A total of 20 movements were recorded for each mouse. The final score was calculated as follows: nonimpaired forelimb movement − impaired forelimb movement/nonimpaired forelimb movement + impaired forelimb movement + both movements.

#### Corner test

The corner test assesses sensorimotor symmetry and was performed as previously described [8]. Ten trials were performed for each mouse, and the percentage of right turns was calculated. Only turns involving full rearing along either board were recorded.

### Tissue Preparation

Four weeks after beginning of BrdU administration or 2 days after beginning of EdU administration, animals were anesthetized with ketamine-xylazine and transcardially perfused with cold PBS followed by 4% PFA in PBS. The brains were post-fixed with 4% PFA and equilibrated in 30% sucrose. They were then sectioned into coronal cryosections measuring 10 µm and mounted directly onto slides for analysis.

### Immunohistochemical Analysis

BrdU immunohistochemistry was performed as described previously [9]. To assess the neuronal phenotype of the BrdU-positive cells, double immunostaining was performed with the following primary antibodies: rat anti-BrdU (1∶200; AbD Serotec, Oxford, UK) and mouse anti-neuronal nuclei (NeuN) (1∶200; Chemicon/Millipore, Temecula, CA, USA). Single Immunostaining was performed with mouse anti-HA (1∶1000, Covonce) and goat anti-doublecortin (DCX) (1∶200, Santa Cruz) after EdU labeling. Anti-GFP (Sigma) antibody was used to detect GFP fluorescence. Following incubation with primary antibodies at 4°C for 24 hours, sections were incubated with secondary antibodies: highly absorbed goat anti-rat Alexa 488 (1∶500, Molecular Probes, Invitrogen), goat anti-mouse Alexa 568 or 488 (1∶500, Molecular Probes, Invitogen) and donkey anti-goat Alexa 568 (1∶500, Molecular Probes, Invitrogen). The antibodies were applied for 1 hour at room temperature. Nuclear DNA was stained with DAPI (1∶200; Sigma-Aldrich).

### EdU Labeling

EdU staining was performed as previously described [10]. Briefly, the sections were incubated with Tris, CuSO_4_, Alexa Fluor 488 Azide (Invitrogen), and ascorbic acid for 30 minutes at room temperature.

### Terminal Transferase-Mediated dUTP Nick End Labeling (TUNEL)

TUNEL assay was performed using the *In Situ* Cell Death Detection Kit, Fluorescein (Roche, Indianapolis, IN, USA), according to the manufacturer’s instructions. Briefly, brain sections were permeabilized in 0.1% Triton X-100 and 0.1% sodium citrate for 15 minutes. Sections were than incubated with the enzyme terminal deoxynucleotidyl transferase and fluorescein-conjugated dUTP at 37°C for 1 hour. Nuclear DNA was stained with DAPI (1∶200; Sigma-Aldrich).

### ELISA

Two days after beginning of EdU administration, animals were sacrificed using guillotine. Immediately thereafter, dissection of the brains was conducted and striatal tissues were separated and cryopreserved in −70°C. Consequently, tissue was thawed and total protein was produced as previously described [6]. Quantification of BDNF levels was conducted using a Brain derived neurotropic factor (BDNF) specific enzyme-linked immunosorbent assay (ELISA) kit (Millipore) according to the manufacturer’s instructions. Protein extracts were loaded on the ELISA plate (in quadruple samples, 25 µg protein in each well). The absorbance at 450 and 570 nm was recorded on a Microplate Reader (Labsystems, Helsinki, Finland). Results were normalized to total amount of protein. Five brains for each group were used for quantification.

### Quantification

For microscopic analysis, we used a Zeiss LSM 510 confocal laser scanning microscope (Carl Zeiss Inc., Thornwood, NJ, USA) or an Olympus BX52TF (Olympus, Lake Success, NY, USA). Regions of interest were defined as a zone with 100 µm width and 700 µm length in the SVZ for counting EdU^+^DCX^+^ and DCX^+^ cells and a box of 500 µm width and length in the ischemic striatum for counting BrdU^+^NeuN^+^, EdU^+^DCX^+^, DCX^+^ and DNA fragmented (TUNEL-stained) cells. Five sections were obtained every 200 µm beginning at a section 0 µm rostral to the bregma, and the results were expressed as the average number per mouse. Five brains for each group were used for quantification.

### Statistical Analysis

Data were analyzed using SPSS software (SPSS, Chicago, IL, USA). Values are presented as mean ± SEM. Differences between groups were compared using two-tailed *t* test or ANOVA followed by Scheffe test. The results were considered significant at *p* < 0.

## Results

### Establishment of Site-specific Wnt3a Overexpressing Mice

**Figure 2:**
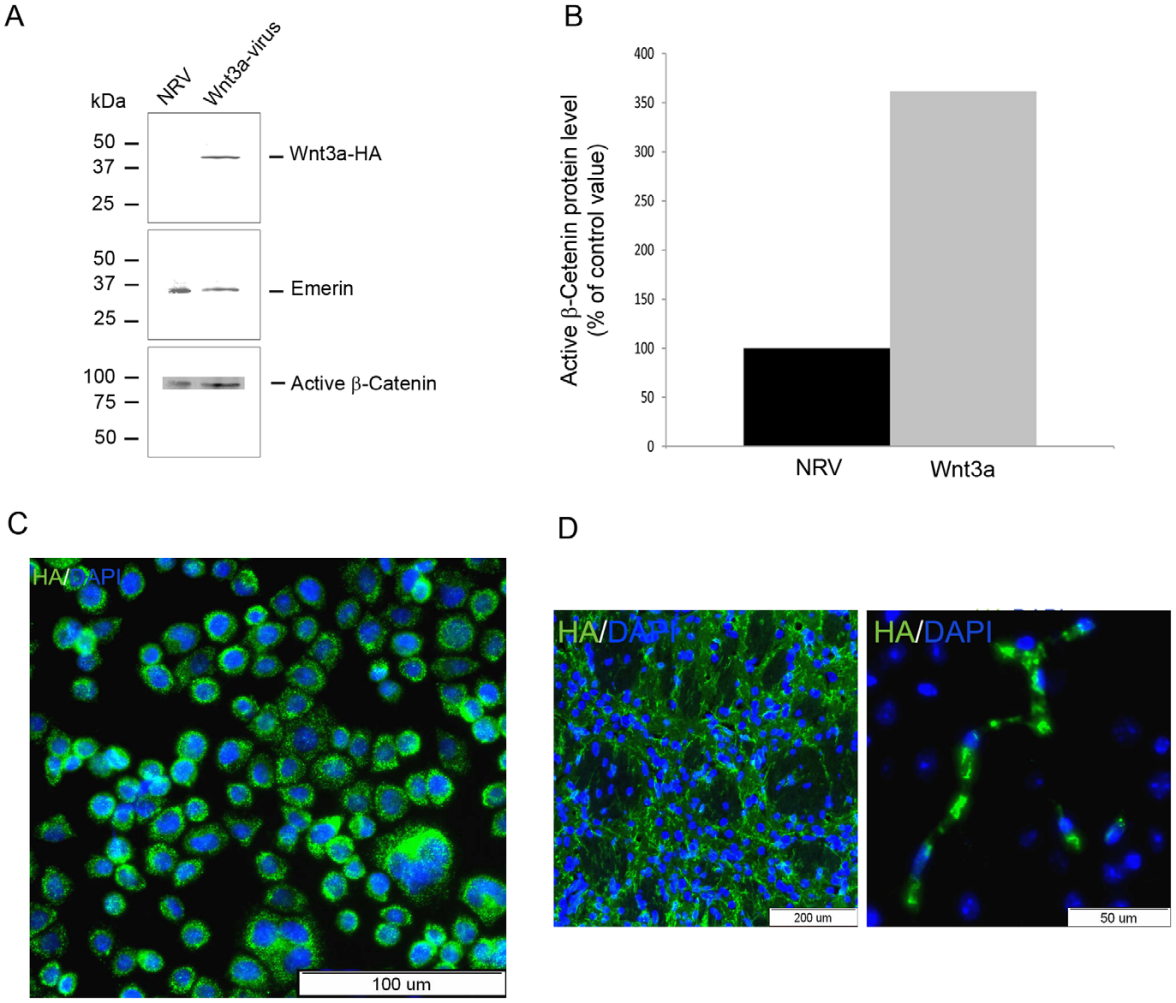
In vitro and in vivo validation of LV-Wnt3a-HA over-expression. A–C. HeLa cells were infected with LV-Wnt3a-HA and confirmation of over-expression was performed using Western blot analysis (A) and immunocytochemistry (C). The ability of Wnt3a-HA to functionally activate Wnt signaling was confirmed by assessing active β-catenin (A) followed by densitometry measurements (B). Cells infected with non-related virus (NRV) were used as control. D. *In vivo* expression of exogenous Wnt3a-HA was detected one month after injection in the striatum.

To generate continuous and site-specific Wnt3a over-expressing mouse models, we designed and generated lentiviruses with the ability to express Wnt3a-HA under the control of the promoter cytomegalovirus. The ability of the Wnt3a-HA lentiviruses to infect cells and induce the expression of Wnt3a-HA was confirmed by infecting HeLa cells. The cell lysates were tested by Western blot analysis, revealing a band of approximately 40 kDa, which correlates with the expected size of Wnt3a (Figure 2A). Such a band did not appear in cells infected with a non-related virus. The blotted membrane was re-probed with anti-emerin antibodies and no significant difference was detected in the amount of the total protein loaded and transferred. To further determine whether increased level of Wnt3a is also reflected in increased Wnt signaling, we measured the protein level of active β-catenin in infected HeLa cells. Cells infected with LV-Wnt3a-HA showed increased levels active β-catenin compared with cells infected with a non-related virus (Figure 2A,B). To visualize the expression of Wnt3a, HeLa cells were plated on plates, infected with the LV-Wnt3a-HA and 48 h later were fixed and immunostained using anti-HA antibody. Wnt3a-HA immunoreactivity was clearly detected in all the cells that were infected (Figure 2C).

The LV-Wnt3a-HA were stereotaxically injected into the striatum of male C57BL/6 mice. The harvested brains were fixed, sliced and immunostained for HA. Viral infected cells showed strong immunoreactivity to HA at the specific loci of injection (Figure 2D).

### Improved Recovery after Focal Ischemic Injury Among Mice Subjected to Wnt3a in the Striatum and SVZ

To study the behavior of Wnt3a over-expression in an ischemic environment, we used the endothelin-1 ischemia model Endothelin-1 is a potent vasoconstrictor that has been used to induce ischemic injury resembling a thrombo-embolic stroke event [11], [12] when injected directly into rodent brain. Before ET-1 exposure, mice were pretreated with the vehicle alone (PBS), LV-GFP or LV-Wnt3a-HA into the striatum or SVZ, and after 5 days reinjected into the striatum with ET-1 to induce ischemic injury.

**Figure 3:**
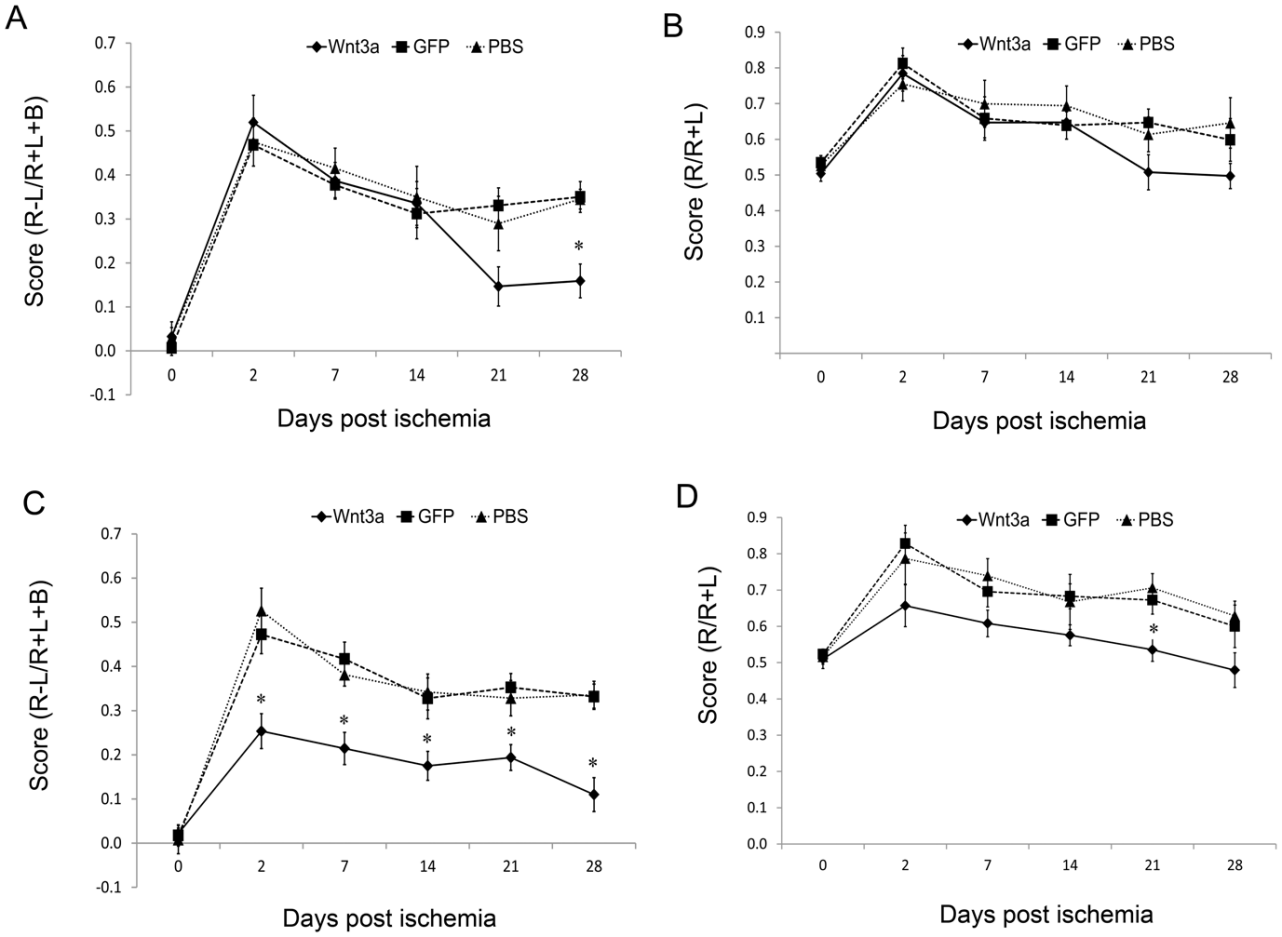
Effect of LV-Wnt3a-HA treatment on functional recovery. A–B. LV-Wnt3a-HA injection into the striatum significantly improved functional performance at 28 days after injury on the cylinder test (A; *p*<0.05) but not on the corner test (B). C–D. LV-Wnt3a-HA treatment into the SVZ significantly improved functional recovery from day 2 after injury on the cylinder test (C; *p*<0.05) and on day 21 after injury on the corner test (D; *p*<0.05). Data are given as mean ± SEM.

The corner and cylinder tests were used to assess motor function. Asymmetry was noted on the cylinder test 2 days after ischemic injury and improved spontaneously over time (Figure 3A,C). In mice injected with LV-Wnt3a-HA into the striatum, improvement was evident from 21 days after injury and was significant at 28 days (Figure 3A). Overall, 68% recovery was achieved on day 28 after injury. The control mice achieved only 30% recovery. Asymmetry was also noted on the corner test 2 days after injury and improved mildly over time (Figure 3B,D). Mice injected with LV-Wnt3a-HA into the striatum did not show an improved performance on the corner test (Figure 3B).

Mice injected with LV-Wnt3a-HA into the SVZ showed a significant improvement in performance on the cylinder test starting 2 days after injury; by day 28, they reached 78% of the optimum performance (Figure 3C). The control mice achieved 32% of the baseline performance. On the corner test, mice injected with LV-Wnt3a-HA into the SVZ showed a significant improvement in performance 21 days after injury (Figure 3D). Therefore, the groups recovered to a similar extent at day 28.

### Effect of Site-specific Expression of Wnt3a on Neurogenesis

**Figure 4:**
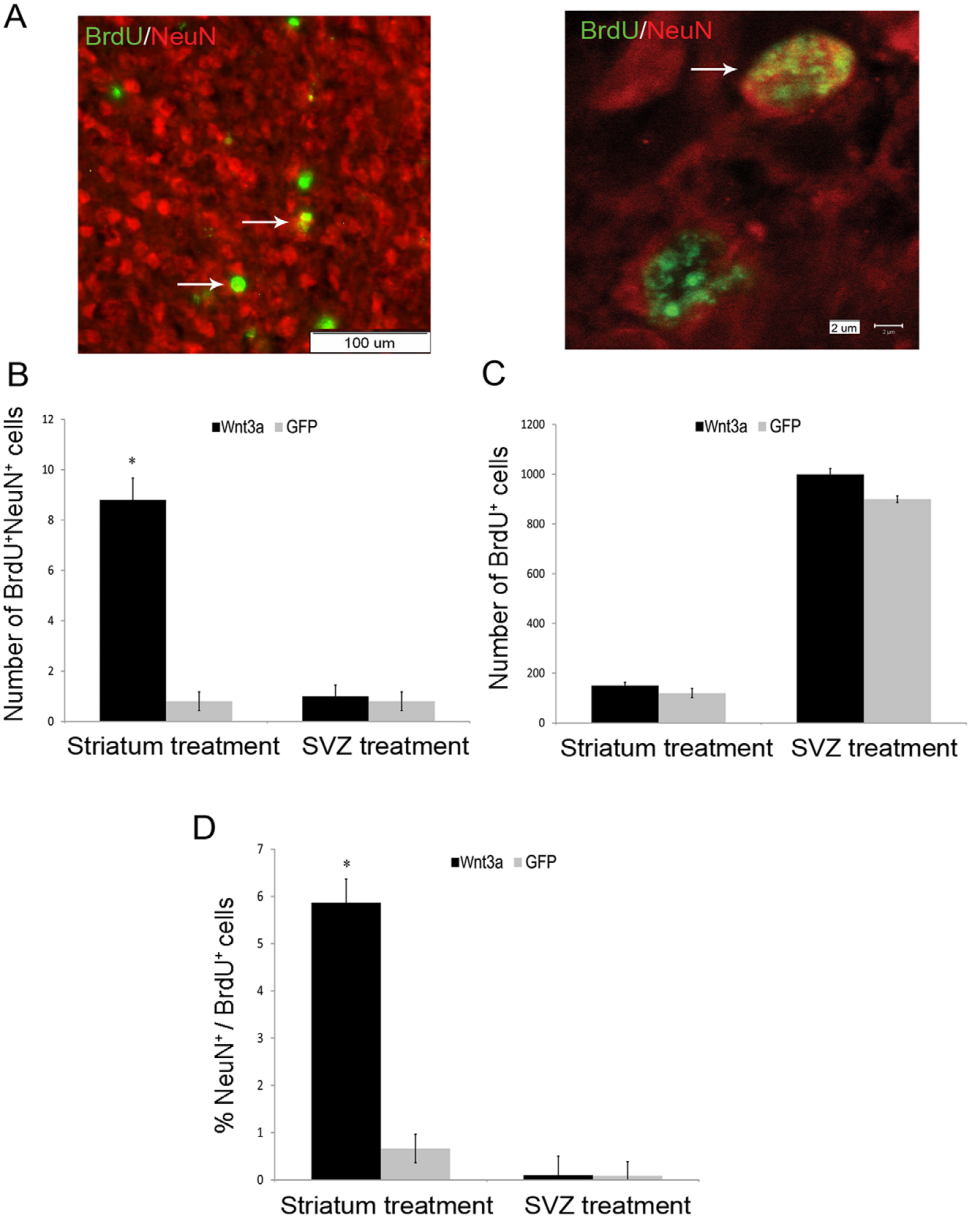
Effect of Wnt3a-HA treatment on neurogenesis 28 days after injury. A. A co-localized BrdU/NeuN cell is shown in the striatum. B–D. Wnt3a-HA injection into the striatum led to a significant increase in the number of newborn neurons in the striatum (*p*<0.01). Treatment with Wnt3a-HA into the SVZ did not change the number of newborn neurons in the striatum. Number of newborn neurons (B), proliferating progenitors (C) and NeuN^+^/BrdU^+^ (D) cells in the striatum and SVZ.

Neurogenesis was assessed by counting the number of BrdU^+^Neun^+^ cells in the striatum on day 28 after injury (Figure 4A). Double-positive cells were detected in the mice treated with LV-Wnt3a-HA injection into the striatum but only seldom in the mice injected with LV-GFP (Figure 4B–D). Double-positive cells were rarely detected in the mice injected with either LV-Wnt3a-HA or LV-GFP into the SVZ (Figure 4B–D). Thus, Wnt3a over-expression in the striatum resulted in increased neurogenesis and improved neurological behavior after 28 days.

**Figure 5:**
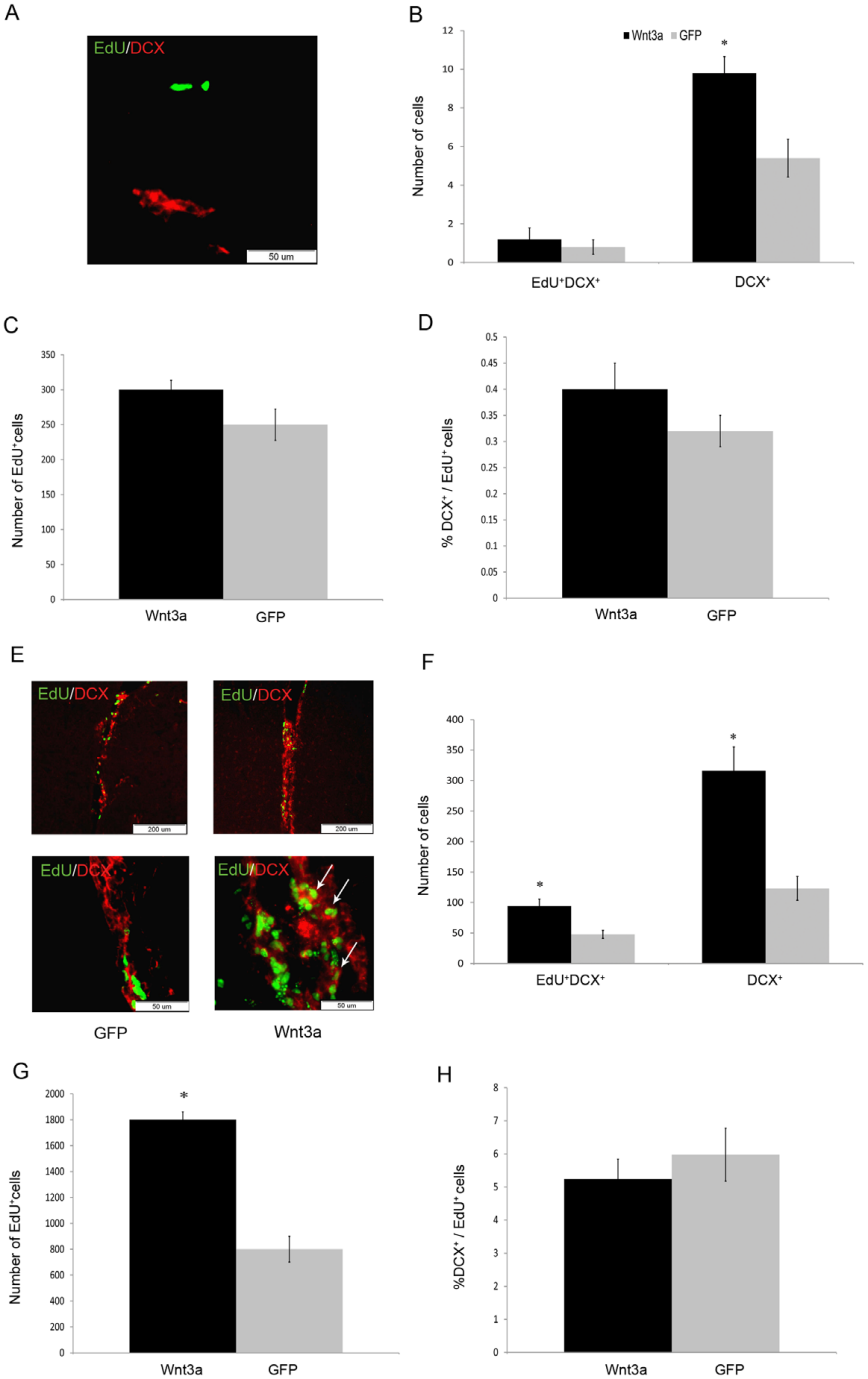
Effect of LV-Wnt3a-HA injection into the SVZ on the proliferation and differentiation of progenitor cells to neuroblasts 2 days after injury. A. EdU^+^ and DCX^+^ cells were detected in the ischemic striatum. B-D. Wnt3a-HA significantly increased the number of DCX^+^ cells in the striatum (*p*<0.01). EdU^+^DCX^+^ cells were hardly detected in the striatum. Number of newborn DCX^+^ (B), proliferating progenitors (C) and DCX^+^/EdU^+^ (D) cells in the striatum. E. EdU^+^ and DCX^+^ cells were found in the SVZ. D-H. Wnt3a-HA significantly increased the number of DCX^+^ and EdU^+^DCX^+^ cells in the SVZ (*p*<0.01). Number of newborn DCX^+^ (F), proliferating progenitors (G) and DCX^+^/EdU^+^ (H) cells in the SVZ.

To assess the cellular changes 2 days after injury in mice treated with LV injection into the SVZ, we counted the number of Edu^+^DCX^+^ cells. Mice injected with LV-Wnt3a-HA showed an increased number of DCX^+^ cells in the ischemic striatum, but not EdU^+^DCX^+^ cells in the striatum (Figure 5A–D). We also found an increased number of DCX^+^ and EdU^+^DCX^+^ cells in the SVZ (Figure 5E–H).

### Effect of Site-specific Expression of Wnt3a in the SVZ on Cell Survival

**Figure 6:**
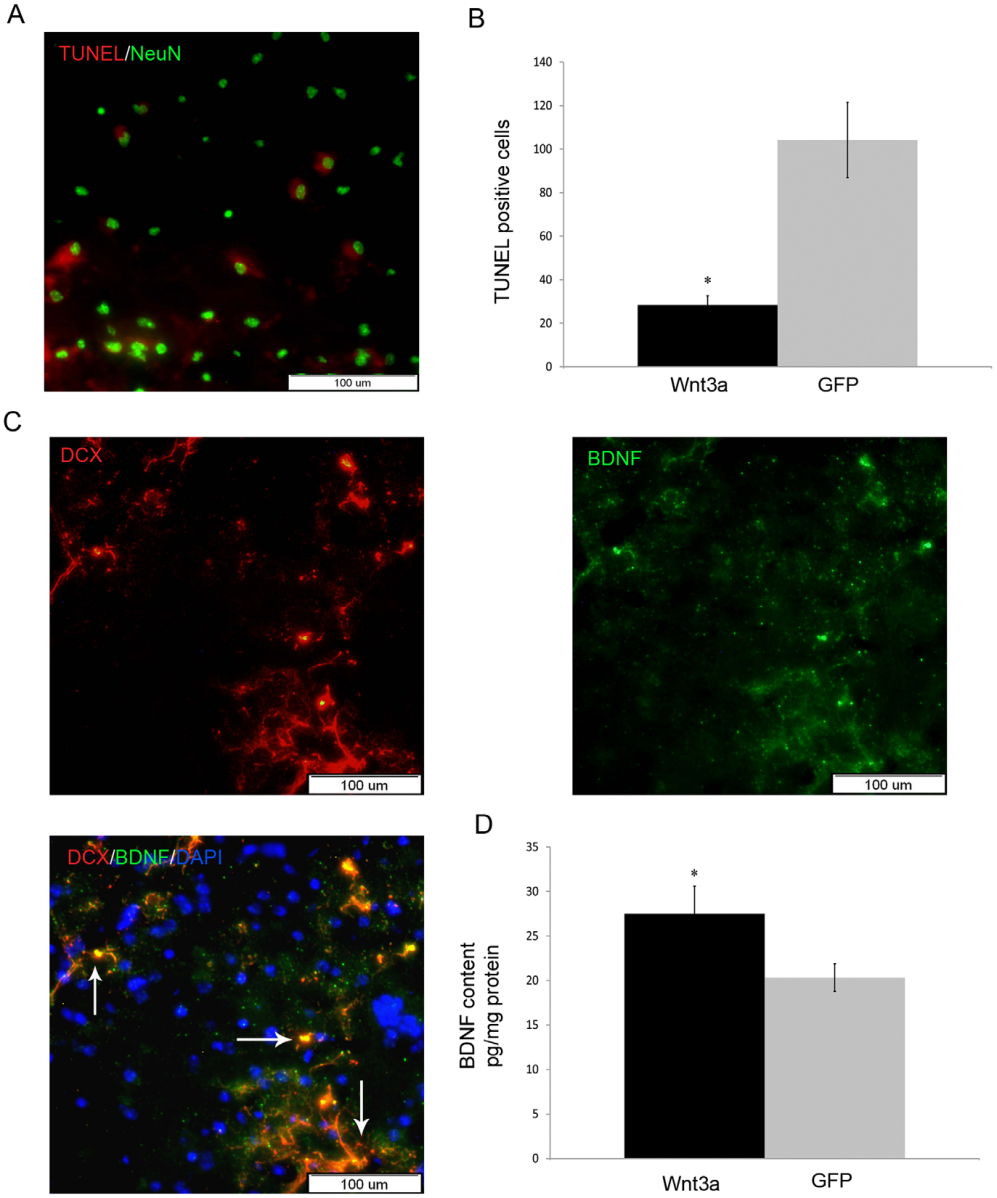
Neuroprotection in the ischemic striatum following LV-Wnt3a-HA injection into the SVZ 2 days after injury. A. DNA strand breaks are labeled by TUNEL staining and NeuN immunohistochemistry in the ischemic striatum. B. Wnt3a-HA significantly reduced the number of DNA fragmented cells in the striatum (*p*<0.05). C. DCX^+^ cells manifest extensive expression of BDNF in the ischemic striatum. D. Quantification of BDNF levels in the striatum using ELISA (*p*<0.05).

Although endothelin-1 resulted in neurological deficit, no distinct brain infarct was found in the striatum 2 days post injury using cresyl violet staining (data not shown). However, widespread neuronal injury was detected in the striatum after ischemic injury using TUNEL staining (Figure 6A). TUNEL-positive cells were positive for the neuronal marker NeuN and very rarely were TUNEL-positive cells colocalized with the astrocyte marker GFAP (data not shown). Previous studies have indicated that mild ischemic injury can result in increased apoptotic cells without evident infarct [13], [14]. Injury-induced apoptosis can be detected from hours to days following induced injury and may contribute to neurological dysfunction.

Wnt3a overexpression in the SVZ was associated with a significant reduction in the number of TUNEL-positive cells 2 days after induction of ischemia (Figure 6B). Quantification revealed a 70% reduction in the density of DNA fragmented cells in the LV-Wnt3a-HA-injected mice. Thus, Wnt3a overexpression in the SVZ resulted in increased neuroblast number in the striatum and neuroprotection after 2 days and accompanied by improvements in neurological behavior.

To determine whether the newly generated neurons can induce a growth-promoting environment, we tested their ability to express BDNF. As shown in Figure 6C, a substantial proportion of the migrating DCX^+^ cells in the striatum, manifested extensive expression of BDNF. Protein lysates of dissected striatal tissues from lentiviral injected mice into the SVZ were analyzed by ELISA. We found 38% higher BDNF levels in the striatum of LV-Wnt3a-HA-injected mice in comparison with mice injected with GFP (Figure 6D).

## Discussion

The present study shows that providing an appropriate environment for the neuronal differentiation of newborn neurons that migrate toward ischemic lesions improves functional recovery after ischemic stroke. We used lentivirus-mediated gene transfer of Wnt3a-HA into the ischemic striatum of a mouse model to enhance neurogenesis. Studies have shown that Wnt3 is expressed in the subgranular zone neurogenic niche by astrocytes and regulates the differentiation of progenitor cells towards neurons [15]. Moreover, Wnt signaling is critically involved in neurogenesis [16]. Inhibition of Wnt signaling in the dentate gyrus reduces the level of newborn neurons and impairs spatial and object recognition in rats. Our study expands these findings, demonstrating that Wnt signaling is able to increase the survival of newly produced neurons also in the non-neurogenic niche. An important question revolves around the functional significance of stroke-induced neurogenesis. At present, it is not completely clear whether the limited numbers of surviving adult-born neurons replace lost cells by integrating appropriately, and whether this improves recovery. However, there are encouraging evidence supports the integration of a small portion of adult-born neurons that migrate to the injured striatum after stroke [17], [18].

The activation of Wnt signaling has been found to offer neuroprotection in models of Alzheimer disease [19]. In our model of focal brain ischemia, Wnt3a did not rescue the neurons in the ischemic environment, although it was overexpressed in the ischemic striatum. The clinical effect was evident only 28 days after injury, indicating that the functional improvement was not attributable to rescue of neurons. However, Wnt signaling is probably involved in the pathogenesis of neuronal death after ischemia, as suggested by reports that Dickkopf-1 (Dkk-1), a Wnt signaling inhibitor, is secreted in the ischemic area of animal models and required for the development of neuronal death [20]. The cell death is associated with induction of apoptosis [21]. Given that high levels of circulating Dkk-1 have been found in patients after acute ischemic stroke [22], these data raise the possibility that, like in Alzheimer disease, rescuing the Wnt signaling pathway might lead to neuroprotection in stroke. This assumption is supported by findings that the administration of lithium, which rescues the Wnt signaling pathway by inhibiting glycogen synthase kinase-3β, was neuroprotective against Dkk-1-induced neurotoxicity [21] and that striatal overexpression of siRNA of beta-catenin, the downstream component of Wnt signaling, caused an enlarged stroke volume [23]. The absence of an association of Wnt3a overexpression in the striatum with neuroprotection might be explained by competition with Dkk-1 secreted from the ischemic tissue. Dkk-1 binds to LRP6, a Wnt receptor on the cell surface, and may thereby interfere with the functional interaction of Wnt with its receptor complex [24]. Thus, it is plausible that effective neuroprotection in the ischemic striatum may be achievable only with downstream Wnt signaling activation.

We hypothesized that the underlying cause for the early improvement of functional performance is related to the proliferation of neural progenitors in the SVZ followed by the migration of neuroblasts into the ischemic striatum. Accordingly, further analysis reveal that overexpression of Wnt3a in the SVZ led to an increase in the number of newborn neurons in the SVZ 2 days after ischemic injury, and this was accompanied by an increased number of newborn neurons that migrated into the ischemic striatum. The attenuation of the brain damage in the mice overexpressing Wnt3a in the SVZ suggests a neuroprotective function of the newborn neurons in the striatum. The new neurons can induce a growth-promoting environment that supports neuroprotection and axonal growth. The latter activity was evidenced by BDNF expression of the new neurons. These findings are in line with previous studies in different disease models showing that neuroprotection is achieved by neural progenitor cells [25], [26] and is probably attributable to the release of trophic factors [27]. Others reported that ablation of neuroblast generation in a stroke model reduced the number of neuroblasts in the ischemic lesion, worsening the clinical outcome and increasing the lesion 24 hours after injury [28].

Following Wnt3a treatment in the SVZ, the number of immature neurons in the striatum increased threefold, without a significant increase in Edu^+^ immature neurons. Thus, the neuroprotective effect is probably derived mostly from cells that proliferate before the ischemic injury. This increased pool of progenitors in the SVZ can apparently be recruited in case of injury.

Previous studies reported that the intraventricular administration of adenoviruses delivering growth factors increased cell proliferation and neurogenesis in the striatum of stroke models [29], [30]. Increased overexpression was noted in the cerebrospinal fluid and other brain tissues, including the cortex and hippocampus. Interestingly, although we observed an increase in the number of immature neurons in the striatum, LV-Wnt3a-HA injection into the SVZ was not associated with an increase in the number of mature neurons. This suggests that the local effect on proliferation in the SVZ is insufficient for neuronal regeneration. Thus, if the environment in the ischemic striatum does not change, the new neurons cannot survive.

Wnt family gene mRNA is detected in the SVZ, but there is no upregulation of these genes after stroke [31]. Nevertheless, endogenous Wnt signaling is probably important for cell proliferation in the SVZ during stroke, considering the decreased proliferation caused by beta-catenin siRNA after middle cerebral artery occlusion [23]. Thus, although Wnt signaling is an important pathway in the SVZ after stroke, it does not naturally upregulate in the reactive SVZ in order to compensate the neuronal damage. Yet, as shown by this study, we can still activate and use the Wnt signaling pathway in our model.

The present study was a proof of concept study to evaluate Wnt3a treatment using the endothelin-1 focal ischemia model. The advantages of this model over the middle cerebral artery occlusion (MCAo) are its simplicity and inherent reliability. A further study using the MCAo model is needed to understand the clinical relevance of these results.

In conclusion, the results of our study show that lentivirus-mediated gene delivery of Wnt3a enhances functional recovery and induces neuroprotection and neurogenesis in the striatum after focal ischemic injury. These findings have important therapeutic implications and should prompt further studies on the use of Wnt signaling to improve functional outcome in patients with stroke.
